# Phonological Assessment Instrument: evidence of construct validity

**DOI:** 10.1590/2317-1782/20232022302en

**Published:** 2023-11-10

**Authors:** Camila Botura, Giovana Sopezack Alves, Ana Carolina Sartori Bernardi, Letícia Pacheco Ribas

**Affiliations:** 1 Universidade Federal de Ciências da Saúde de Porto Alegre - UFCSPA - Porto Alegre (RS), Brasil.; 2 Departamento de Fonoaudiologia, Universidade Federal de Ciências da Saúde de Porto Alegre - UFCSPA - Porto Alegre (RS), Brasil.

**Keywords:** Validation Study, Reproducibility of Results, Language Tests, Speech Articulation Tests, Speech, Speech Sound Disorder, Child, Speech, Language and Hearing Sciences

## Abstract

**Purpose:**

To investigate evidence of construct validity for a Phonological Assessment Instrument for Brazilian Portuguese, based on the diagnostic data generated by its application from contrastive analysis and speech severity.

**Methods:**

The sample consisted of 176 children, aged between five to nine years old. They were evaluated with the Phonological Assessment Instrument and then classified as having Speech Sound Disorder or in typical phonological development, comparing these results to the criteria described for the disorder in the DSM-5. The search for evidence of construct validity relied on the agreement between the two assessment methods while applying the Kappa Coefficient. To differentiate between groups, Student's t-test was used for independent samples. We sought to investigate the instrument indexes using the Receiver Operating Characteristic Curve statistics to obtain values for area, cut-off point, sensitivity, specificity, accuracy, and positive and negative predictive value.

**Results:**

The instrument showed agreement and significant differentiation between the classifications. As for the performance parameters, it shows a cut-off point for diagnosis with results equal to or greater than 96.17%, an excellent area under the curve, as well as satisfactory percentages for the other analyses investigated.

**Conclusion:**

The data indicated evidence for the construct validity of the Phonological Assessment Instrument, presenting a useful and valid contribution to the arsenal of clinical assessment and research involving the diagnosis of Speech Sound Disorder and, with its accuracy result, contributed to the properties of performance of instruments used in Speech, Language and Hearing Sciences.

## INTRODUCTION

Phonological Disorder (PD)^^1^^ is a linguistic disorder that is manifested by a deviation in the phonological ability of a given child compared to what is expected for that age group. This disorder does not have a defined etiology, however, it affects the phonological level of language organization, presenting performance difficulties concerning phonemes and/or syllabic structures of the language being acquired^(1)^. The criteria for clinical diagnosis referred to in the Diagnostic and Statistical Manual of Mental Disorders (DSM-5)^(2)^ include (a) persistent difficulty in speech production; (b) age greater than four years; (c) auditory thresholds within normal limits; (d) absence of neurological alterations or evident organic causes; (e) normal intellectual abilities; (f) speech understanding ability; and, (g) expressive language without alterations regarding lexicon and syntax.

The set of signs listed enables the clinical diagnosis of PD, highlighting the speech assessment, which allows the detailing of the linguistic organization and the understanding of the child's phonetic and phonological acquisition by the speech therapist^(3)^. In this sense, through the observation of the variability of production, it becomes possible to analyze the phonemes that have already been acquired, those that have not been, and those that are in the acquisition process^(1)^.

In speech evaluation, the analysis of the phonological system is recommended by verifying the production of sounds and comparing them with the expected patterns of the target language^(3)^. Thus, it is necessary that the instruments for such assessments go through psychometric studies carried out with speakers of the mother tongue to measure their skills and parameters.

Currently, the literature on assessment measures has been advancing within Brazilian speech therapy^(4,5)^. There are instruments established in clinical use (such as Children's Phonological Assessment - AFC^(6)^ and ABFW - Children's Language Test - Phonology^(7)^) that are widely used by professionals in the area. They help in the diagnostic and speech therapy process, in addition to providing parameters for several studies. However, despite such prestigious instruments and the development of new tests^(8-10)^, there are still gaps in the development of a tool as a scientifically proven gold standard in the field of oral language in Brazilian Portuguese (BP). As a result, accurate diagnostic identification is hampered, hindering the evolution of both clinical activity and scientific knowledge on the subject.

Studies on psychometrics are unanimous in considering reliability and validity as the main parameters for instrument legitimacy^(5,11,12)^. Reliability is a test’s ability to consistently reproduce its result, indicating aspects of coherence, stability, and accuracy. Validity concerns the aspect of the measure being coherent with the competence that is intended to be evaluated, that is, measuring what it is proposed to measure^(12,13)^. In this sense, it is possible to make an analogy that reliability responds to the percentage of correct answers- if something is correct and what is its intensity, while validity responds to the reality of the measurement- if something is true and how it was based^(14)^.

In order to determine validity, we can separate it into two of its concepts: content validity and construct validity. The first evaluates the degree to which the content of an instrument adequately reflects its objective, that is, how much the sample of items represents the domain of the content. For such validation, the material must be analyzed by expert judges in the area. The second, the focus of this study, verifies whether the test set represents the elaborated theoretical construct, analyzing through the instrument itself whether the observed behaviors measure the desired latent trait^(15)^.

Considering these parameters, to understand the construct it is necessary to understand the process that is constituted as the cause of its behavior. The construct of a test is assumed to be true since it allows measuring the action that manifests a latent trait of the mental process^(14)^. Thus, the present study aimed to investigate evidence of construct validity for a Phonological Assessment Instrument for BP, based on diagnostic data generated by its application based on contrastive analysis and the degree of speech severity.

## METHODS

The research corresponds to an observational, cross-sectional, controlled, descriptive, and quantitative study, whose data were used for the construct validity of the Phonological Assessment Instrument (IAF). This study was approved by the Research Ethics Committee of a federal university under number 5.045.533.

### Sample

The sample size was calculated considering 25% of PD, according to the estimated prevalence of the diagnosis for the child population^(9)^. To determine a Kappa coefficient of 0.80, indicating substantial agreement, and for a significance of 5% and power of 80%, the result was a minimum of 165 children for the representative sample.

The *corpus* of this study consists of data from 176 children, between five and nine years old, from a public school in the city of Porto Alegre, selected from a data bank with 219 evaluations. Data from children who had auditory, lexical, and syntactic alterations in relation to expressive language, evident neurological and/or organic, intellectual and/or cognitive alterations, language comprehension and school difficulties, history of neuropsychomotor delay, and/or intercurrences in pregnancy or childbirth. Those with characteristics of typical phonological development and those diagnosed with PD were included. Such categorization was obtained from the results of language assessments carried out with the entire *corpus*, as well as reports in interviews with those responsible. All parents or guardians signed a Free and Informed Consent Term and Authorization for Audio Use; and, in the case of children over 7 years old, they also signed a Term of Assent.

### Speech Assessment Instrument - IAF

The IAF^^2^^ is a software designed to evaluate the child's speech sound system efficiently, thoroughly, and optimally. The instrument was elaborated with 123 words, belonging to children's vocabulary, extracted from popular children's stories, easily represented in an image or photo, and of the noun type, with an image corresponding to each lexical item. The items were carefully selected so that the words included all consonant phonemes in all syllabic positions in BP, with five occurrences of each phoneme and syllabic position, totaling 235 phonemic possibilities. The collection of the child's speech should occur from the naming of each of the images, by observing the illustrations or photographs, which takes approximately 10 minutes for the application. The evaluator must record the audio of the speech collection, and later, listen and observe the children's elicitations and register the information to the software. This process takes between 10 and 30 minutes, depending on the evaluator's practice and skill. After inserting data referring to the production of each target phoneme in the instrument, the results are automatically generated. They are expressed in descriptive and quantitative reports by the degree of speech severity^^3^^ , contrastive analysis, phonological processes, and change in distinctive features.

### Procedures

The assessment of how the measured variables represent the instrument's construct requires a qualitative theoretical analysis^(14)^ with the search for quantitative evidence of agreement. However, studies on the subject point to the absence of an oral language assessment instrument considered the gold standard in BP, hindering the possibility of comparative calculations^(5,11)^. From this, the choice was made to use the reference described in the criteria for Speech Disorder in the DSM-5^(2)^, circumventing this impediment with the justification of being the manual that not only helps in the diagnosis of PD, but how it was prepared to be the standard resource in the definition of disorders that affect the mind and emotions^(2,16)^.

An evaluation includes the use of convergent and discriminant relationships^(13)^. Applied to this study, the convergent analysis aims to ascertain whether the instrument has a high degree of agreement compared to another with the same outcome, that is, whether the diagnosis obtained with the IAF agrees with the reference described in the criteria for Speech Disorder of the DSM-5^(2)^. As for the discriminant ratio, which evaluates the ability to distinguish compared to different target populations; in this case, if the group with typical phonological development obtained by the IAF is the same as those who do not fulfill the criteria determined by the DSM-5^(2-11)^.

Speech collections (n = 179) in audio format were analyzed using the IAF software by three evaluators - undergraduate students in speech therapy and trained in phonetic analysis. These were blinded in relation to the sample. In case of disagreement, a consensus was sought among the evaluators and, finally, a fourth evaluation was performed by a specialized speech therapist.

The final result of the diagnosis of PD for each of the children was organized in spreadsheets. For the IAF analysis, the following were considered: contrastive analysis, with acquisition definition value>75%^(1)^, and; the degree of speech severity, according to the calculation of Percentage of Correct Consonants - Revised (PCC-R)^(17)^, which considers the percentage of correct productions without considering articulatory distortions as errors. In the analysis using the DSM-5^(2)^ framework, the diagnostic criteria for Speech Disorder were fulfilled based on information from the language assessments already considered in the sample selection for this research study. Finally, each of the instruments resulted in a diagnosis, associating 0= 'Typical Phonological Development', 1= 'Phonological Disorder'.

### Data analysis

The results were presented through frequencies and percentages. For the statistical analysis, the SPSS software version 28 for Windows was used.

Cohen's Kappa Coefficient^(18)^ was applied to measure the agreement between the two assessment methods (IAF and DSM-5^(2)^) for the diagnosis of PD since it corrects the value for the frequency with which they may agree by chance. An investigation was also carried out using Student's t-test^(19)^ for independent samples, to verify whether the IAF results differed between the classifications according to the DSM-5^(2)^.

To describe its classification performance, the most recommended analytical method is the Receiver Operating Characteristic Curve (ROC)^(5,13,20)^, using the area under the curve (AUC) indicators, and the cut-off point, according to the index of Youden^(20)^, with values obtained through the graduation of scores from the degree of severity (PCC-R). For general comparisons of test performance, sensitivity, specificity, and accuracy values of the instrument were highlighted. To substantiate its clinical applicability, the calculation of the positive predictive value (PPV) was used as an analysis for convergent validity, as well as the negative predictive value (NPV) used for divergent validity. Estimates of Cohen's Kappa coefficient, sensitivity, specificity, and AUC are presented with a 95% confidence interval (95% CI).

## RESULTS

The search for evidence for the construct validity of this study consisted of two analyses: agreement and difference between the results and assessment of the instrument's indexes. The general degree of agreement in the diagnosis classification between the two methods resulted in a coefficient of 0.759 (95% CI 0.612 to 0.905, p<0.001). This value is described as a moderate level (0.6≤ Kappa ≥0.79), interpreted as adequate for confidence in the results^(18)^.

Table 1 shows the mean values obtained by the IAF for each classification of the groups according to the DSM-5^(2)^ criteria, performed with Student's t-test for independent samples. The results reveal that the average of the group with typical phonological development was higher than the group with a diagnosis of PD. This shows that the IAF scores differentiate children with alterations in the phonological system from those who do not. It is noteworthy that the high variability in the standard deviation value found for the diagnosis of PD may represent the difference in the children's phonological system profiles. There may be many phonemes not yet acquired (severe degree of PD) or few phonemes not yet acquired(medium-grade PD).

**Table 1:** IAF results according to DSM-5 classifications

**DSM-5**	***n***	**Mean**	**SD**	**p-value**
Typical Phonological Development	124	**97.75**	2.85	<0.001*
PD Diagnosis	55	**87.29**	9.38	

* Significant values (p≤0.05)

**Caption:** IAF = Phonological Assessment Instrument; DSM-5 = Diagnostic and Statistical Manual of Mental Disorders, 5th edition; PD = Phonological disorder; *n* = Number of children classified by the IAF; SD = Standard deviation

As for the evidence for the evaluation of the IAF performance parameters, the ROC Curve analysis was used (Table 2). The cutoff point found for the instrument's classification corresponds to the simultaneous optimization of sensitivity and specificity and is based on the percentages of the degree of speech severity. Thus, values greater than or equal to 96.17% correspond to the categorization of 'Typical Phonological Development' and lower findings to 'PD’

**Table 2:** Area of the ROC Curve for IAF results in children with PD

AUC	p-value	Lower limit	Upper limit	IAF Cutoff Point (<=)
0.945	<0.001*	0.912	0.977	96.17%


* Significant values (p≤0.05)

**Caption:** IAF = Phonological Assessment Instrument; PD = Phonological disorder; AUC = Area under the curve; ROC curve = Values of area under the curve

Regarding the size of the effect of the ROC Curve, shown in Table 2, the most used indicator is the AUC, which indicates the degree of differentiation for the test diagnoses. For the IAF, the AUC result is identified as excellent, since the value is greater than 0.9, very close to 1, which would be ideal^(20)^.

Based on the aforementioned cutoff value, the results are shown in Table 1. The classifications obtained by the instruments are presented separately at the respective ends of 'Total'. With the objective of comparing the efficiency of the IAF in relation to the DSM-5^(2)^ (gold standard reference), in the center the categorizations are indicated in a crossed manner, allowing the visualization of the diagnoses for PD and for typical phonological development determined by the standard, including their proportion by the IAF.

The data presented in Table 1 allow the calculations for the analyses in Table 3, which show IAF performance indexes. In bold are the results in which there is agreement between the two methods, considered as true classifications. The other values are seen as false diagnoses, since they are determined by the IAF, but disagree with the reference. The sensitivity and specificity measures highlighted describe the instrument's ability to detect the correct classification for an already known population.

**Table 3:** IAF performance indexes

*Índex*	%	Confidence Interval (CI)
*Sensitivity*	89.1%	CI 95%: 78.17% to 94.9%
*Specificity*	89.5%	CI 95%: 82.89% to 93.77%
*True-positive*	**27.7%**	-
*True-negative*	**62.0%**	-
*False-positive*	7.3%	-
*False-negative*	3.4%	-
*Positive predictive value (PPV)*	79.0%	-
*Negative Predictive Value (NPV)*	94.9%	-
*Accuracy*	**89.4%**	-
*Estimated prevalence (IAF)*	34.6%	-
*Actual prevalence (DSM-5)*	30.7%	-

**Caption:** IAF = Phonological Assessment Instrument; DSM-5 = Diagnostic and Statistical Manual of Mental Disorders, 5th edition; 95% CI = 95% confidence interval

The VPP and NPV analyses describe the proportion of correct IAF conclusions within each classification in an unknown population. Such results are evidence for convergent and discriminant validity, respectively. The convergent predicts the proportion of true PD diagnoses determined by the IAF, while the discriminant verifies the proportion of true cases of typical phonological development. Thus, the IAF presents predictive values that confirm sensitivity and specificity (Table 3).

Regarding accuracy, which is the probability that the test provides correct conclusions regardless of the diagnostic category, the IAF has a significant index, as seen in Table 3. This measure infers the overall power of the instrument, in addition to being applied for comparisons with other instruments.

## DISCUSSION

The development of speech sounds represents the phonological domain in the mental process of language acquisition. It is necessary to evaluate the precision in the production of these sounds for an investigation of the organization of phonemes^(3)^. Thus, the theoretical and technical foundations assume the construct of the instrument as true and adequate, since the ability to measure the latent trait of phonological acquisition and systematization is explored from the speech behavior^(11,14)^. This study presents evidence for the construct validity of the IAF, which confirms adequate indicators for its use as an instrument in the phonological assessment of children.

Construct validity, in addition to the theoretical basis, also requires empirical confirmation. The exclusive use of the DSM-5^(2)^ defines criteria and allows an analysis that refers only to the observation of the flow of speech and the indication of whether or not there are alterations in the sounds and/or syllables spoken. On the other hand, the instrumental measure with the collection of isolated words complements this process^(21,22)^. It details the phonological profile in a more agile, systematic, detailed, and precise way, guaranteeing significant clinical data for the treatment and evolution. In the presentation of the results in Chart 1, the value of false diagnoses based on the DSM-5 is justified by the aforementioned reasons.

**Chart 1:** IAF x DSM-5 results ratio

		*DSM-5*		Total for IAF (*n*)
		PD Diagnosis *(n)*	Typical Phonological Development *(n)*	
*IAF*	PD Diagnosis (<=96.17%)	**49**	13	62
	Typical Phonological Development (>96.17%)	6	**111**	117
Total for DSM-5 (*n*)		55	124	179

**Caption:** IAF = Phonological Assessment Instrument; PD = Phonological disorder; DSM-5 = Diagnostic and Statistical Manual of Mental Disorders, 5th edition; *n* = Number of children classified

In this sense, it is important to assess the classification agreement between the two methods (IAF and DSM-5^(2)^) and it was concluded that, even as complementary tools, when applied separately, they still present a good correlation. The level at which the IAF is related to the results of the DSM-5^(2)^ is within the standards established as acceptable, both for health care and for clinical research^(18)^. This means that the test set represents consistency in its application, confirming that one of the objectives was satisfactorily achieved.

As expected, the average results of the group with typical phonological development were lower than those of the group with diagnosis (Table 1). This shows that the results of the IAF are sensitive to identifying children with or without PD, meeting its main objective as an instrument. The INFONO^(10)^ software, which evaluates phonology, also presents statistically significant results to aid in the differential diagnosis, but it is not yet available to the public.

The IAF parameters presented in this study serve to analyze its performance as a classification model. Both the cutoff point found - which is related to the high sensitivity and specificity - and the degree of differentiation for the test diagnoses obtained a result with values close to the ideal^(20)^. None of the studies on oral language instruments in speech therapy research uses the ROC curve, despite being the requested statistical estimator^(5,13,23)^. Only one study was found with a similar objective, population, and methodology. However, it evaluated only sensitivity, specificity, and the cutoff point in the use of the PCC-R calculation in another reference test^(17)^. From the results obtained, the values are equivalent; however, the cutoff point determined for the IAF is higher and, consequently, possibly more sensitive and more specific for the diagnosis of PD.

Another study that also has the same population, with the delimitation of cases and analysis circumstances, despite a different methodology (it does not use the ROC curve), evaluated the sensitivity and specificity parameters of the Terdaf instrument^(24)^. This test was designed to track speech disorders in children and its performance results are lower than those of the IAF, as it is less sensitive and specific, as well as having lower predictive values.

The sensitivity and specificity indexes, observed in Table 3, are essential for theoretical comparison between tests. However, the predictive values describe evidence of relevance for clinical practice. They point to the proportion of categorization of the IAF among an unknown population, which underlies the power of the instrument in helping the diagnostic decision^(25)^.

The discriminant validity was defined with the negative predictive value and stands out, pointing out that the instrument well identifies the population that does not have the diagnosis, that is, with typical phonological development. This finding is homogeneous in descriptions of the test properties, both in studies carried out with BP speakers^(17,24)^ and with other languages^(21,26)^. Furthermore, these studies conclude that there is no significant difference when the age variable applied to picture naming tests is evaluated. For this reason, the IAF data were not designed to separate participants based on such criteria.

Regarding the accuracy analysis, recent reviews on the subject indicate that no phonological assessment test investigates such data and that they are necessary to verify the overall quality of the instrument's measurement^(5,13)^. The relevance of the findings of the present research study is emphasized since the measure is significant for the IAF and adds the performance properties of the instruments used in the area. This, therefore, demonstrates that the instrument is a useful and valid contribution to the arsenal of clinical and research evaluation involving the diagnosis of PD.

Finally, it is highlighted that the instrument is structured for BP speakers in general; however, the data used are exclusively with the phonological profile of children from a single public school in the city of Porto Alegre/RS. Just as it should be noted that the IAF software was developed to assess any age group. However, this validation is constituted by a sample of children aged from five years old, which respects the period of stabilization of the phonological acquisition and the criteria for the diagnosis of PD described in the DSM-5^(2)^. Therefore, it is recommended that studies addressing the consequences of the test apply it to samples with regional variability, types of schools, and age groups. Furthermore, the IAF provides support for future studies to explore comparisons between it and other phonological assessment tests.

## CONCLUSION

The set of data found indicates good evidence for the construct validity of the Phonological Assessment Instrument - IAF. In addition, it demonstrates being able to achieve objective, systematic, and relevant results both in the evaluation and in the analysis. This study fulfills a stage of instrument construct validation. It should be noted that all other steps for the psychometric process of the IAF are being carried out and disseminated in parallel, including the establishment of standards of reliability with safety.

## Footnotes

1 Phonological Disorder is in the diagnosis that ASHA calls Speech Sound Disorder and that DSM-5 names Speech Disorder. However, the term Phonological Disorder was chosen because both in ASHA and DSM-5, it is exposed in a general way. There, it encompasses both articulation and phonological disorders in the same denomination, being the responsibility of the professional to differentiate the diagnoses and in this study, what is being distinguished specifically is Phonological Disorder.

2 Psychometric studies on the instrument's properties of content validity, response-process, and reliability are in the process of being published.

3 The degree of speech severity is guided by the classification of the PCC-R calculation, which is the percentage of the number of correct consonants produced in the child's speech in relation to the total number of consonants possible in the instrument.
