# Supplementary material for: Phonological Assessment Instrument: evidence of construct validity
Source: Codas. 2023 Nov 10;36(1):e20220302. doi: 10.1590/2317-1782/20232022302en (PMC10715583; doi:10.1590/2317-1782/20232022302en)
Supplement: Translation

# Translation: Instrumento de Avaliação Fonológica: evidências de validade de construto

**DOI:** 10.1590/2317-1782/20232022302pt

**Authors:** Camila Botura, Giovana Sopezack Alves, Ana Carolina Sartori Bernardi, Letícia Pacheco Ribas

**Affiliations:** 1 Universidade Federal de Ciências da Saúde de Porto Alegre - UFCSPA - Porto Alegre (RS), Brasil.; 2 Departamento de Fonoaudiologia, Universidade Federal de Ciências da Saúde de Porto Alegre - UFCSPA - Porto Alegre (RS), Brasil.

**Keywords:** Estudo de Validação, Testes de Linguagem, Testes de Articulação da Fala, Fala, Transtorno Fonológico, Criança, Fonoaudiologia

## Abstract

**Objetivo:**

Investigar evidências de validade de construto para um Instrumento de Avaliação Fonológica para o Português Brasileiro, baseadas nos dados de diagnóstico gerados por sua própria aplicação a partir da análise contrastiva e do grau de severidade de fala.

**Método:**

A amostra foi composta por dados de 176 crianças, com idades entre cinco até nove anos. Foram avaliadas pelo Instrumento de Avaliação Fonológica e classificadas em com transtorno fonológico ou em desenvolvimento fonológico típico, comparando tais resultados aos critérios para o transtorno no DSM-5. A busca por evidências da validade de construto contou com a concordância entre os dois métodos de avaliação, aplicando o Coeficiente Kappa. Para a diferenciação entre os grupos, utilizou-se o teste t de Student para amostras independentes. Buscou-se a investigação dos índices do instrumento pela estatística da Curva de Receiver Operating Characteristic para obter valores de área, ponto de corte, sensibilidade, especificidade, acurácia, valor preditivo positivo e negativo.

**Resultados:**

O instrumento apresentou concordância e diferenciação significativa entre as classificações. Quanto aos parâmetros de desempenho, exibe ponto de corte para diagnóstico com resultados iguais ou maiores do que 96,17%, excelente valor de área sob a curva, assim como percentuais satisfatórios para as outras análises investigadas.

**Conclusão:**

O conjunto de dados encontrados indicam evidências para validade de construto do Instrumento de Avaliação Fonológica, apresentando uma contribuição útil e válida ao arsenal de avaliação clínica e de pesquisa envolvendo diagnóstico de Transtorno Fonológico e, com seu resultado de acurácia, contribuiu as propriedades de desempenho dos instrumentos utilizados na Fonoaudiologia.

## INTRODUÇÃO

O Transtorno Fonológico (TF)^^1^^ é uma desordem linguística que se manifesta por um desvio na capacidade fonológica de determinada criança em comparação ao esperado para aquela faixa etária. Esse transtorno não possui etiologia definida, contudo, afeta o nível fonológico da organização da linguagem, apresentando dificuldade de desempenho com relação aos fonemas e/ou estruturas silábicas da língua em aquisição^(1)^. Os critérios para o diagnóstico clínico referidos no Manual Diagnóstico e Estatístico de Transtornos Mentais (DSM-5)^(2)^ englobam: (a) dificuldade persistente para produção de fala; (b) idade superior a quatro anos; (c) limiares auditivos dentro dos padrões de normalidade; (d) ausência de alterações neurológicas ou causas orgânicas evidentes; (e) habilidades intelectuais normais; (f) capacidade de compreensão da fala; e, (g) linguagem expressiva sem alterações com relação ao léxico e à sintaxe.

O conjunto de sinais listados viabiliza o diagnóstico clínico de TF, destacando a avaliação de fala, que permite o detalhamento da organização linguística e a compreensão da aquisição fonética e fonológica da criança pelo fonoaudiólogo^(3)^. Nesse sentido, através da observação da variabilidade de produção, torna-se possível analisar os fonemas que já foram adquiridos, os que não foram, e, os que estão em processo de aquisição^(1)^.

Na avaliação da fala preconiza-se a análise do sistema fonológico por meio da verificação da produção dos sons e da comparação com os padrões esperados da língua alvo^(3)^. Dessa forma, faz-se necessário que os instrumentos para tais avaliações passem por estudos psicométricos realizados com falantes da língua materna para mensurar suas competências e parâmetros.

Atualmente, a literatura sobre medidas de avaliação vem avançando dentro da fonoaudiologia brasileira^(4,5)^. Existem instrumentos consagrados no uso clínico (como por exemplo: Avaliação Fonológica da Criança - AFC^(6)^ e ABFW - Teste de Linguagem Infantil - Fonologia^(7)^) muito utilizados pelos profissionais da área que auxiliam no processo diagnóstico e fonoterapêutico, além de fornecerem parâmetros para diversos estudos. No entanto, apesar de tais instrumentos prestigiados e embora exista o desenvolvimentos de novos testes^(8-10)^, ainda há lacunas na elaboração de uma ferramenta como padrão-ouro cientificamente comprovada na área da linguagem oral em Português Brasileiro (PB). Por causa disso, tem-se prejuízos na identificação diagnóstica precisa, dificultando a evolução tanto da atividade clínica, quanto do conhecimento científico sobre o tema.

Os estudos sobre psicometria são unânimes em considerar a fidedignidade e a validade como principais parâmetros para a legitimidade de instrumentos^(5,11,12)^. A fidedignidade é a capacidade de um teste em reproduzir seu resultado de forma consistente, indicando aspectos sobre a coerência, estabilidade e precisão. A validade diz respeito ao aspecto da medida ser coerente com a competência que se almeja avaliar, ou seja, medir o que se propõe a medir^(12,13)^. Nesse sentido, é possível fazer uma analogia de que a fidedignidade responde ao percentual de acertos, se algo está correto e qual sua intensidade, enquanto a validade responde à realidade da medida, se algo é verdadeiro e como foi fundamentado^(14)^.

A fim de determinar a validade, podemos separá-la em dois de seus conceitos: validade de conteúdo e de construto. A primeira avalia o grau em que o conteúdo de um instrumento reflete adequadamente o objetivo dele, isto é, o quanto a amostra de itens de fato representa o domínio do conteúdo; para tal validação é necessário que o material seja analisado por juízes especialistas na área. Já a segunda, foco deste estudo, verifica se o conjunto do teste representa o construto teórico elaborado, analisando pelo próprio instrumento se os comportamentos observados medem o traço latente almejado^(15)^.

Considerando esses parâmetros, para compreender o construto se faz necessário entender o processo que é constituído como a causa do seu comportamento. O construto de um teste se assume verdadeiro uma vez que permite mensurar a ação que manifesta um traço latente do processo mental^(14)^. Assim, o presente estudo teve como objetivo investigar evidências de validade de construto para um Instrumento de Avaliação Fonológica para o PB, baseadas nos dados de diagnóstico gerados por sua própria aplicação a partir da análise contrastiva e do grau de severidade de fala.

## MÉTODO

A pesquisa corresponde a um estudo observacional, transversal controlado, descritivo e quantitativo, cujos dados foram utilizados para a validade de construto do Instrumento de Avaliação Fonológica (IAF). Este trabalho foi aprovado pelo Comitê de Ética em Pesquisa (CEP) da Universidade Federal de Ciências da Saúde de Porto Alegre (UFCSPA) sob parecer número 5.045.533.

### Amostra

O tamanho amostral foi calculado considerando 25% de TF, conforme estimativa de prevalência do diagnóstico para a população infantil^(9)^. Para determinar um coeficiente Kappa de 0,80, indicando boa concordância e para significância de 5% e poder de 80%, o resultado foi de minimamente 165 crianças para a amostra representativa.

O *corpus* deste estudo é constituído por dados de 176 crianças, entre cinco anos até nove anos de idade, de uma escola pública da cidade de Porto Alegre, selecionadas de um banco com 219 avaliações. Foram excluídos os dados de crianças que possuíam alterações auditivas, lexicais e sintáticas com relação à linguagem expressiva, neurológicas e/ou orgânicas evidentes, intelectuais e/ou cognitivas, dificuldades compreensivas de linguagem, escolares, histórico de atraso neuropsicomotor e/ou de intercorrências em gravidez ou parto. Foram incluídos aqueles com características de desenvolvimento fonológico típico e os com diagnóstico de TF. Tal categorização foi obtida a partir dos resultados das avaliações de linguagem realizadas com todo o *corpus,* assim como dos relatos em entrevista de com os responsáveis. Todos os pais ou os responsáveis assinaram Termo de Consentimento Livre e Esclarecido (TCLE) e Autorização para Uso de Áudio; e, no caso de crianças acima de 7 anos, essas também assinaram Termo de Assentimento.

### Instrumento de Avaliação de Fala - IAF

O IAF^^2^^ é um *software* delineado para avaliar o sistema de sons da fala infantil de forma eficiente, minuciosa e otimizada. O instrumento foi elaborado com 123 palavras, pertencem ao vocabulário infantil, extraídas de histórias infantis populares, facilmente representadas em imagem ou foto e são do tipo substantivo, com uma imagem correspondente a cada item lexical. Os itens foram criteriosamente selecionados de forma que as palavras contemplassem todos os fonemas consonantais em todas as posições silábicas do PB, com cinco ocorrências de cada fonema e posição silábica, totalizando 235 possibilidades fonêmicas. A coleta da fala da criança deve ocorrer a partir da nomeação de cada uma das imagens, pela observação das ilustrações ou fotografias, que leva aproximadamente 10 minutos para a aplicação. O avaliador deve gravar o áudio da coleta de fala, e posteriormente, ouvir e observar as elicitações das crianças e registrar as informações no *software*. Tal processo leva em torno de 10 a 30 minutos, dependendo da prática e habilidade do avaliador. Após a inserção dos dados referentes à produção de cada fonema alvo no instrumento, os resultados são gerados automaticamente e expressos em relatórios descritivos e quantitativos por: grau de severidade de fala^^3^^ , análise contrastiva, processos fonológicos e mudança de traços distintivos.

### Procedimentos

A avaliação de como as variáveis medidas representam o construto do instrumento exige uma análise teórica qualitativa^(14)^ com a busca por evidências quantitativas de concordância. No entanto, os estudos sobre o assunto apontam para a ausência de um instrumento de avaliação de linguagem oral considerado como padrão-ouro em PB, dificultando a possibilidade de cálculos comparativos^(5,11)^. A partir disso, a escolha foi pelo uso do referencial descrito nos critérios para Transtorno de Fala do DSM-5^(2)^, contornando esse impeditivo com a justificativa de ser o manual que não só auxilia no diagnóstico de TF, mas como foi elaborado para ser o recurso padrão na definição de transtornos que afetem a mente e as emoções^(2,16)^.

Assim como tal avaliação inclui o uso das relações convergente e discriminante^(13)^. Aplicada a este estudo, a análise convergente visa averiguar se o instrumento possui uma elevada concordância comparado com outro de mesmo desfecho, isto é, se o diagnóstico obtido com o IAF concorda com o referencial descrito nos critérios para Transtorno de Fala do DSM-5^(2)^. Já a relação discriminante que avalia a capacidade de distinção comparada a diferentes populações-alvo; neste caso, se o grupo com desenvolvimento fonológico típico obtido pelo IAF é o mesmo dos que não preenchem os critérios determinados pelo DSM-5^(2-11)^.

As coletas de fala (n = 179) em formato de áudio foram analisadas com a utilização do *software* do IAF por três avaliadoras - graduandas em fonoaudiologia e treinadas para análise fonética. Essas foram cegadas em relação à amostra. Em caso de discordância, buscou-se o consenso entre as avaliadoras e, por fim, uma quarta avaliação foi realizada por uma fonoaudióloga especializada.

O resultado final do diagnóstico de TF de cada uma das crianças foi organizado em planilhas. Para a análise do IAF foram consideradas: a análise contrastiva, com valor de definição de aquisição>75%^(1)^, e; o grau de severidade de fala, conforme o cálculo de Percentual de Consoantes Corretas - Revisado (PCC-R)^(17)^, que considera a porcentagem de produções corretas sem contabilizar distorções articulatórias como erro. Já na análise pelo referencial DSM-5^(2)^, os critérios do diagnóstico para Transtorno de Fala foram preenchidos a partir das informações das avaliações de linguagem já consideradas na seleção da amostra desta pesquisa. Por fim, cada um dos instrumentos resultou em um diagnóstico, relacionando 0= ‘Desenvolvimento Fonológico Típico’, 1= ‘Transtorno Fonológico’.

### Análise dos dados

Os resultados foram apresentados através de frequências e percentuais. Para a análise estatística foi utilizado o *software* SPSS versão 28 para Windows.

Foi aplicado o Coeficiente Kappa de Cohen^(18)^ para mensurar a concordância entre os dois métodos de avaliação (IAF e DSM-5^(2)^) de diagnóstico de TF, uma vez que corrige o valor pela frequência com que podem concordar pelo acaso. Realizou-se, também, uma investigação através do teste *t* de Student^(19)^ para amostras independentes, com o intuito de verificar se os resultados do IAF diferenciavam entre as classificações de acordo com o DSM-5^(2)^.

Para descrever seu desempenho classificatório, o método analítico mais recomendado é a Curva de Receiver Operating Characteristic (ROC)^(5,13,20)^, usando os indicadores de área sob a curva (AUC), e ponto de corte, segundo o índice de Youden^(20)^, com valores obtidos através da graduação de escores do grau de severidade (PCC-R). Para comparações gerais do desempenho do teste, foram destacados os valores de sensibilidade, especificidade e acurácia do instrumento. Para fundamentação da sua aplicabilidade clínica, o cálculo de valor preditivo positivo (VPP) foi utilizado como análise para validade convergente, tal como o de valor preditivo negativo (VPN) usado para validade divergente. As estimativas do coeficiente Kappa de Cohen, sensibilidade, especificidade e a AUC foram apresentadas com intervalo de confiança de 95% (IC95%).

## RESULTADOS

A busca por evidências para validade de construto deste trabalho foi constituída por duas análises: concordância e diferença entre os resultados e avaliação dos índices do instrumento. O grau geral de concordância na classificação do diagnóstico entre os dois métodos obteve como resultado o coeficiente de 0,759 (IC 95% 0,612 a 0,905, p<0,001). Esse valor é descrito como nível moderado (0,6≤ Kappa ≥0,79), interpretado como adequado para confiança nos resultados^(18)^.

A Tabela 1 demonstra os valores de médias obtidas pelo IAF para cada classificação dos grupos de acordo com os critérios do DSM-5^(2)^, realizados com o teste *t* de Student para amostras independentes. Os resultados revelam que a média do grupo com desenvolvimento fonológico típico foi superior ao grupo com diagnóstico de TF. Isso evidencia que os escores do IAF diferenciam crianças com alterações no sistema fonológico daquelas que não as apresentam. Vale ressaltar que a alta variabilidade no valor de desvio-padrão encontrada para o diagnóstico de TF pode representar a diferença nos perfis de sistema fonológicos das crianças, nos quais pode haver muitos fonemas ainda não adquiridos (TF de grau severo) ou poucos fonemas ainda não adquiridos (TF de grau médio).

**Tabela 1:** Resultados do IAF de acordo com as classificações do DSM-5

**DSM-5**	**n**	**Média**	**DP**	**p-valor**
Desenvolvimento Fonológico Típico	124	**97,75**	2,85	<0,001*
Diagnóstico de TF	55	**87,29**	9,38	

* Valores significantes (p≤0,05)

**Legenda:** IAF = Instrumento de Avaliação Fonológica; DSM-5 = Manual Diagnóstico e Estatístico de Transtornos Mentais, 5ª edição; TF = Transtorno fonológico; *n =* Número de crianças classificadas pelo IAF; DP = Desvio padrão

Quanto às evidências para a avaliação dos parâmetros de desempenho do IAF, utilizou-se a análise pela Curva ROC (Tabela 2). O ponto de corte encontrado para a classificação do instrumento corresponde à otimização simultânea da sensibilidade e especificidade e é baseado nos percentuais de grau de severidade de fala. Assim, os valores maiores ou iguais do que 96,17% correspondem à categorização de ‘Desenvolvimento Fonológico Típico’ e achados menores a de ‘Transtorno Fonológico’.

**Tabela 2:** Área da Curva ROC para resultados do IAF em crianças com TF

AUC	p-valor	Limite Inferior	Limite Superior	Ponto de Corte IAF (<=)
0,945	<0,001*	0,912	0,977	96,17%

* Valores significantes (p≤0,05)

**Legenda:** IAF = Instrumento de Avaliação Fonológica; TF = Transtorno fonológico; AUC = Área sob a curva;

Curva ROC = Valores de área sob a curva

Em relação à dimensão do efeito da Curva ROC, exibida na Tabela 2, o indicador mais empregado é a AUC que aponta o grau de diferenciação para os diagnósticos do teste. Para o IAF, o resultado de AUC é identificado como excelente, uma vez que o valor é maior que 0,9, bem próximo à 1, que seria o ideal^(20)^.

A partir do valor de ponto de corte supracitado são exibidos os resultados no Quadro 1. As classificações obtidas pelos instrumentos são apresentadas separadamente nas extremidades respectivas de ‘Total’. Com o objetivo de comparar a eficiência do IAF em relação ao DSM-5^(2)^ (referencial de padrão-ouro), no centro são apontadas as categorizações de forma cruzada, permitindo a visualização dos diagnósticos para TF e para desenvolvimento fonológico típico determinados pelo padrão, incluindo a proporção dos mesmos pelo IAF.

**Quadro 1:** Relação de resultados IAF x DSM-5

		*DSM-5*		Total para IAF (*n*)
		Diagnóstico TF *(n)*	Desenvolvimento Fonológico Típico *(n)*	
*IAF*	Diagnóstico TF (<=96,17%)	**49**	13	62
	Desenvolvimento Fonológico Típico (>96,17%)	6	**111**	117
Total para DSM-5 (*n*)		55	124	179

**Legenda:** IAF = Instrumento de Avaliação Fonológica; TF = Transtorno fonológico; DSM-5 = Manual Diagnóstico e Estatístico de Transtornos Mentais, 5ª edição; *n* = Número de crianças classificadas

Os dados apresentados no Quadro 1 permitem os cálculos para as análises da Tabela 3, que exibem índices de desempenho do IAF. Em negrito estão os resultados em que há concordância entre os dois métodos, considerado como classificações verdadeiras. Já os outros valores são vistos como diagnósticos falsos, uma vez que são determinados pelo IAF, mas discordam do referencial. Destacam-se as medidas de sensibilidade e especificidade que descrevem a capacidade do instrumento de detectar a classificação correta para uma população já conhecida.

**Tabela 3:** Índices de desempenho do IAF

*Índice*	%	Intervalo de confiança (IC)
*Sensibilidade*	89,1%	IC 95%: 78,17% a 94,9%
*Especificidade*	89,5%	IC 95%: 82,89% a 93,77%
*Verdadeiro-positivo*	**27,7%**	-
*Verdadeiro-negativo*	**62,0%**	-
*Falso-positivo*	7,3%	-
*Falso-negativo*	3,4%	-
*Valor preditivo positivo (VPP)*	79,0%	-
*Valor preditivo negativo (VPN)*	94,9%	-
*Acurácia*	**89,4%**	-
*Prevalência estimada (IAF)*	34,6%	-
*Prevalência real (DSM-5)*	30,7%	-

**Legenda:** IAF = Instrumento de Avaliação Fonológica; DSM-5 = Manual Diagnóstico e Estatístico de Transtornos Mentais, 5ª edição; IC 95% = Intervalo de Confiança 95%

As análises de VPP e VPN descrevem a proporção de conclusões corretas do IAF dentro de cada classificação em uma população desconhecida. Tais resultados são evidências para as validades convergente e discriminante, respectivamente. A convergente prevê a proporção de verdadeiros diagnósticos de TF determinados pelo IAF, enquanto a discriminante verifica a proporção de verdadeiros quadros de desenvolvimento fonológico típico. Dessa forma, o IAF apresenta valores preditivos que confirmam sensibilidade e especificidade (Tabela 3).

Em relação à acurácia, que é a probabilidade de o teste fornecer conclusões corretas seja qual for a categoria diagnóstica, o IAF apresenta um índice significativo, como visto na Tabela 3. Essa medida infere o poder geral do instrumento, além de ser aplicada para comparações com outros instrumentos.

## DISCUSSÃO

O desenvolvimento dos sons da fala representa o domínio fonológico no processo mental de aquisição de linguagem. Faz-se necessário avaliar a precisão na produção desses sons para uma investigação da organização dos fonemas^(3)^. Assim, as fundamentações teóricas e técnicas assumem o construto do instrumento como verdadeiro e adequado, uma vez que a partir do comportamento da fala explora-se a capacidade de mensurar o traço latente da aquisição e sistematização fonológica^(11,14)^. Esta pesquisa apresenta as evidências para a validade de construto do IAF, que confirma indicadores adequados para a sua utilização como instrumento na avaliação fonológica em crianças.

A validade de construto, além do embasamento teórico, também requer confirmações empíricas. O uso exclusivo do DSM-5^(2)^ define critérios e permite uma análise do que se refere apenas à observação do fluxo de fala e a indicação se há ou não alterações nos sons e/ou sílabas faladas. Por outro lado, a medida instrumental com a coleta de palavras isoladas complementa esse processo^(21,22)^, pois detalha o perfil fonológico de forma mais ágil, sistemática, minuciosa e precisa, garantindo dados clínicos significativos para o tratamento e o guia da evolução. Na apresentação dos resultados do Quadro 1, o valor de diagnósticos falsos a partir do DSM-5 é justificado pelos motivos supracitados.

Nesse sentido, é importante avaliar a concordância da classificação entre os dois métodos (IAF e DSM-5^(2)^) e concluiu-se que, mesmo como ferramentas complementares, quando aplicadas separadamente, ainda assim apresentam boa correlação. O nível em que o IAF relaciona-se com os resultados do DSM-5^(2)^ se encontra dentro dos padrões estabelecidos como aceitáveis, tanto para a assistência à saúde, como para a pesquisa clínica^(18)^. Isso significa que o conjunto do teste representa consistência em sua aplicação, confirmando que um dos objetivos foi alcançado de modo satisfatório.

Como era esperado, os resultados médios do grupo com desenvolvimento fonológico típico foram inferiores aos do grupo com diagnóstico (Tabela 1). Isso evidencia que os resultados do IAF são sensíveis para identificar crianças com TF ou sem, atendendo seu principal objetivo como instrumento. O *software* INFONO^(10)^, que avalia fonologia, também apresenta resultados com significância estatística para auxiliar no diagnóstico diferencial, mas ainda não está disponibilizado para o público.

Os parâmetros do IAF, apresentados neste estudo, servem para análise do seu desempenho como modelo classificatório. Tanto o ponto de corte encontrado - que se relaciona com a alta sensibilidade e especificidade -, como o grau de diferenciação para os diagnósticos do teste obtiveram um resultado com valores próximos ao ideal^(20)^. Nenhum dos estudos sobre instrumentos de linguagem oral nas pesquisas em fonoaudiologia utiliza a Curva ROC, apesar de ser o estimador estatístico solicitado^(5,13,23)^. Apenas um estudo foi encontrado com similar objetivo, população e metodologia; entretanto avaliando somente sensibilidade, especificidade e ponto de corte na utilização do cálculo de PCC-R em outro teste referencial^(17)^. Dos resultados obtidos, os valores são equivalentes; porém o ponto de corte determinado para o IAF é mais alto e, por consequência, possivelmente mais sensível e mais específico para o diagnóstico de TF.

Outro estudo que também possui a mesma população, com delimitação de casos e circunstância da análise, apesar de diferente metodologia (não utiliza a Curva de ROC), avaliou os parâmetros de sensibilidade e especificidade do instrumento Terdaf^(24)^. Esse teste foi projetado para o rastreamento de alterações de fala em crianças e seus resultados de desempenho são inferiores aos do IAF, por ser menos sensível e específico, assim como possuir menores valores preditivos.

Os índices de sensibilidade e especificidade, observados na Tabela 3, são essenciais para comparação teórica entre testes. No entanto, os valores preditivos descrevem evidências de relevância para a prática clínica, já que apontam a proporção de categorização do IAF em meio a uma população desconhecida, o que fundamenta o poder do instrumento em auxiliar a decisão diagnóstica^(25)^.

A validade discriminante foi definida com o valor preditivo negativo e se destaca, apontando que o instrumento identifica bem a população que não possui o diagnóstico, ou seja, com desenvolvimento fonológico típico. Esse achado é homogêneo em descrições das propriedades dos testes, tanto em estudos realizados em falantes de PB^(17,24)^ quanto com outras línguas^(21,26)^. Ademais, esses estudos concluem que não há diferença significativa quando é avaliada a variável de idade aplicada às provas de nomeação de figuras; por esse motivo, os dados do IAF não foram delineados para separação dos participantes a partir de tais critérios.

Em relação à análise sobre acurácia, as revisões recentes na temática indicam que nenhum teste de avaliação fonológica investiga tais dados e que eles são necessários para verificar a qualidade geral da medida do instrumento^(5,13)^. Ressalta-se a relevância dos achados da presente pesquisa, uma vez que a medida é evidentemente significativa para o IAF e acrescenta as propriedades de desempenho dos instrumentos utilizados na área. Isso, portanto, demonstra que o instrumento é uma contribuição útil e válida ao arsenal de avaliação clínica e de pesquisa envolvendo diagnóstico de TF.

Por fim, destaca-se que o instrumento é estruturado para falantes de PB em geral; no entanto, os dados utilizados são exclusivamente com o perfil fonológico de crianças de uma única escola pública do município de Porto Alegre/RS. Assim como cabe ressaltar que o *software* do IAF foi desenvolvido para avaliar qualquer faixa etária; contudo esta validação é constituída por uma amostra de crianças com idade a partir de cinco anos, o que respeita o período de estabilização da aquisição fonológica e os critérios para o diagnóstico de TF descritos no DSM-5^(2)^. Recomenda-se, então, que pesquisas abordando as consequências do teste o apliquem em amostras com variabilidade regional, tipos de escola e faixa etária. Ademais, o IAF apresenta sustentação para que próximos estudos explorem comparações entre ele e outros testes de avaliação fonológicas.

## CONCLUSÃO

O conjunto de dados encontrados indica boas evidências para validade de construto do Instrumento de Avaliação Fonológica - IAF. Além disso, demonstra alcançar resultados objetivos, sistemáticos e relevantes tanto na avaliação, quanto na análise. Este estudo cumpre uma etapa da validação de construto do instrumento. Salienta-se que todas as demais etapas para o processo psicométrico do IAF estão sendo paralelamente realizadas e divulgadas, inclusive com o estabelecimento dos padrões de fidedignidade com segurança.

## Footnotes

1 O Transtorno Fonológico está no diagnóstico que a ASHA denomina de Transtorno dos Sons da Fala e que o DSM-5 nomeia de Transtorno da Fala. Porém, preferimos optar por Transtorno Fonológico, pois tanto na ASHA quanto no DSM-5, ele é exposto de forma geral, englobando tanto os transtornos de articulação quanto fonológico na mesma denominação, sendo responsabilidade do profissional diferenciar os diagnósticos e neste estudo estamos falando especificamente de Transtorno Fonológico.

2 Estudos psicométricos sobre as propriedades do instrumento de validade de conteúdo, processo-resposta e confiabilidade estão em processo de publicação.

3 O grau de severidade de fala é guiado pela classificação do cálculo de PCC-R, sendo ele a porcentagem de número de consoantes corretas produzidas na fala da criança em relação ao número total de consoantes possíveis pelo instrumento.
